# Nanoscale evidence of erbium clustering in Er-doped silicon-rich silica

**DOI:** 10.1186/1556-276X-8-39

**Published:** 2013-01-21

**Authors:** Etienne Talbot, Rodrigue Lardé, Philippe Pareige, Larysa Khomenkova, Khalil Hijazi, Fabrice Gourbilleau

**Affiliations:** 1 Groupe de Physique des Matériaux (GPM), Université et INSA de Rouen, UMR CNRS 6634, Normandie Université, Av. de l’Université, BP 12, Saint Etienne du Rouvray, 76801, France; 2 Centre de Recherche sur les Ions, les Matériaux et la Photonique (CIMAP), CEA/CNRS/ENSICAEN/UCBN, 6 Bd. Maréchal Juin, Caen Cedex 4, 14050, France

**Keywords:** Erbium, Silicon, Nanocrystallites, Nanoclusters, Sputtering, Atom probe tomography, Photoluminescence

## Abstract

Photoluminescence spectroscopy and atom probe tomography were used to explore the optical activity and microstructure of Er^3+^-doped Si-rich SiO_2_ thin films fabricated by radio-frequency magnetron sputtering. The effect of post-fabrication annealing treatment on the properties of the films was investigated. The evolution of the nanoscale structure upon an annealing treatment was found to control the interrelation between the radiative recombination of the carriers via Si clusters and via 4*f* shell transitions in Er^3+^ ions. The most efficient 1.53-*μ*m Er^3+^ photoluminescence was observed from the films submitted to low-temperature treatment ranging from 600°C to 900°C. An annealing treatment at 1,100°C, used often to form Si nanocrystallites, favors an intense emission in visible spectral range with the maximum peak at about 740 nm. Along with this, a drastic decrease of 1.53-*μ*m Er^3+^ photoluminescence emission was detected. The atom probe results demonstrated that the clustering of Er^3+^ ions upon such high-temperature annealing treatment was the main reason. The diffusion parameters of Si and Er^3+^ ions as well as a chemical composition of different clusters were also obtained. The films annealed at 1,100°C contain pure spherical Si nanocrystallites, ErSi_3_O_6_ clusters, and free Er^3+^ ions embedded in SiO_2_ host. The mean size and the density of Si nanocrystallites were found to be 1.3± 0.3 nm and (3.1± 0.2)×10^18^ Si nanocrystallites·cm^−3^, respectively. The density of ErSi_3_O_6_ clusters was estimated to be (2.0± 0.2)×10^18^ clusters·cm^−3^, keeping about 30% of the total Er^3+^ amount. These Er-rich clusters had a mean radius of about 1.5 nm and demonstrated preferable formation in the vicinity of Si nanocrystallites.

## Background

Silicon-based photonics is a fast growing field of semiconductor nanoscience. A part of this area focuses on the realization of integrated optoelectronic devices (such as light planar waveguide amplifier, light-emitting diodes, lasers, ..) to overcome the interconnect bottleneck for Si-based integrated circuits. In this regard, the use of optical interconnection is the most promising. Among the different strategies, the most considered for Si-based telecommunication are (1) doping of silica fibers with Er^3+^ ions which offered the emission at the standard telecommunication wavelength (1.53 *μ*m) and (2) incorporation of quantum-confined Si nanoclusters (Si-ncs) or nanocrystallites (Si-NCs) in such doped fibers, favoring an enhancement of Er-effective excitation cross section. Both these approaches fully exploit the individual properties of Si-ncs (Si-NCs) and rare-earth ions [1,2].

It was already demonstrated that Si-nc/SiO_2_ interface affects significantly not only the properties of the Si-ncs themselves, but also the optical activity of Er^3+^ ions coupled with Si-ncs [1,3,4]. It was shown that a thin 0.8-nm sub-stoichiometric interface between the Si-nc and the SiO_2_ host plays a critical role in the Si-nc emission [5,6]. Furthermore, numerous studies allowed the determination of the main mechanism of the interaction between the Si-ncs and the neighboring Er^3+^ ions [1,2,7]. Along with the effect of structural environment of both Er^3+^ ions and Si-ncs on their individual properties, it has also been observed that very small Si-ncs, even amorphous, allow an efficient sensitizing effect towards Er^3+^ ions. However, the efficiency of this process depends on the separating distance between Si-ncs and rare-earth ions [7-9]. Critical interaction distances were found to be about 0.5 nm [7,9,10].

In spite of the significant progress in the investigation of the excitation processes in Er-doped Si-rich SiO_2_ materials, some issues are still debatable, such as the spatial location of optically active Er^3+^ ions with regard to Si-ncs. Another aspect, which may control the optical properties, is the distribution of Er dopants in the film, i.e., either these ions are uniformly distributed or they form some agglomerates [11]. Thus, mapping the Si and Er^3+^ distributions in Er-doped Si-rich SiO_2_ films as well as the investigation of the evolution of these distributions versus fabrication conditions and post-fabrication processing are the key issues to manage the required light-emitting properties of such systems.

Up to now, high-resolution and energy-filtered transmission electron microscopies were the only techniques offered a direct visualization of Si and Er distributions [11-13]. Nevertheless, other indirect techniques, such as fluorescence-extended X-ray absorption fine-structure spectroscopy [14-16] or X-ray photoelectron spectroscopy [17], have evidenced that the amount of Er clusters in Er-doped Si-rich SiO_2_ films depends strongly on the preparation conditions or annealing temperature. We have recently demonstrated the feasibility of atom probe tomography (APT) analysis of Si-rich SiO_2_ systems, giving its atomic insight [18,19]. With the benefit of this expertise, the purpose of this paper is to perform a deep analysis of Er-doped Si-rich SiO_2_ thin films by means of APT experiments to understand the link between the nanoscale structure of the films and their optical properties. The distributions of Si and Er^3+^ ions in as-grown films were investigated. The evolutions of chemical composition of the films upon annealing treatment, the formation of Si-ncs, and the redistribution of Er^3+^ ions were studied with the aim of finding the way to control the microstructure at the atomic scale and to optimize light-emitting properties of the Er-doped Si-rich SiO_2_ system.

## Methods

### Sample fabrication

Er-doped Si-rich SiO_2_ (Er-SRSO) layers were grown by radio-frequency (RF) magnetron-sputtering technique. For the APT experiments, the deposition was performed on an array of p-doped Si(100) posts (5 *μ*m in diameter and 100 *μ*m in height). This method, already used in previous works, allows a simple procedure for atom probe sample preparation [20]. For optical experiments, the layers were grown on standard p-type (100) Si wafers in the same deposition run. The film fabrication approach comprises the co-sputtering of Er_2_O_3_, SiO_2_, and Si targets in pure argon plasma on substrate kept at 500°C. The Er content and the Si excess were independently controlled through the RF power applied on the corresponding cathode. More details on the fabrication processes can be found in other works [12,21]. The thickness of the Er-SRSO layer was 200 nm. The concentration of Er^3+^ ions in the sample was 1×10^21^at./cm^3^, while the Si excess was about 5 at.% [21]. To study the effect of post-fabrication treatment on structural and optical properties of the layers, each sample was divided into several parts. One of them was kept as a reference for the ‘as-deposited’ state. The others were submitted to an annealing treatment in conventional furnace in constant nitrogen flow to study the phase separation, the Si-nc formation, the recovering of the defects, and thus, the enhancement of Er emission. The samples were annealed at 600°C for 10 h, 900°C for 1 h, and 1,100°C for 1 h. The annealing time for each temperature corresponds to optimal conditions, giving rise to the highest photoluminescence of the Er^3+^ ions.

### Atom probe tomography

Among the various analytical techniques, atom probe tomography is one of the most promising when atomic scale resolution, three-dimension reconstruction, and quantitative chemical characterization are required [22,23]. The recent improvement of this technique with the implementation of femtosecond laser pulses [24] allowed to enlarge the variety of materials to be studied. Thus, an atomic observation of photonic, solar cells, magnetic semiconductor, or nanoelectronic devices is now available [18,19,25-28]. The Er-SRSO film with the shape of a tiny needle, required for APT analyses, was prepared using a focused ion beam annular milling procedure. The details of this standard procedure are reported in another work [20]. In order to prevent the layer of interest from Ga damages and/or amorphization during the sample processing, a 300-nm-thick layer of Cr was pre-deposited on the top of the sample. Films were then ion-milled into sharp tips with an end radius close to 30 nm. A low-accelerating voltage (2 kV) was used for the final stage in order to avoid Ga implantation and sample amorphization. The APT used in this work is the CAMECA (CAMECA SAS, Gennevilliers Cedex, France) laser-assisted wide-angle tomographic atom probe. The experiments were performed with samples cooled down to 80 K, with a vacuum of (2 to 3)×10^−10^ mbar in the analysis chamber and with ultraviolet (*λ*=343 nm) femtosecond (350 fs) laser pulses. The laser energy was fixed at 50 nJ/pulse focused onto an approximately 0.01-mm^2^ spot.

To identify the clusters, the algorithm described hereafter was applied. Each step of this identification comprises the placement of a sphere (sampling volume) over one atom of the volume investigated and the estimation of the local composition of the selected elements by counting atoms within this sphere. If the composition exceeds a given threshold, the atom at the center of the sphere is associated to a cluster. If the composition is lower than the threshold, the atom at the center of the sphere belongs to the matrix. The sphere is then moved to the next atom, and this procedure is applied again to estimate the composition and to compare it with the threshold value. This approach was used for all the atoms of the volume to identify those belonging either to the clusters or to the matrix. In this paper, a threshold of 75% of Si and 5% of Er was used to identify pure Si nanoclusters and Er-rich regions with a sphere radius of 1 nm.

### Photoluminescence study

The photoluminescence (PL) properties of the samples were examined using the 476-nm excitation line delivered by an Innova 90C coherent Ar^+^ laser (Coherent Inc., Santa Clara, CA, USA). The pumping at 476 nm, which is nonresonant for Er^3+^ ions, was always used to ensure that Er^3+^ excitation was mediated by the Si-based sensitizers. The Er^3+^ PL spectra in the 1.3- to 1.7-*μ*m spectral range were measured at room temperature by means of a Jobin Yvon (HORIBA Jobin Yvon Inc., Edison, NJ, USA) 1-m single-grating monochromator coupled to a North Coast germanium detector (North Coast Scientific Co., Santa Rosa, CA, USA) cooled with liquid nitrogen. The Si-nc PL properties were investigated in the 550- to 1,150-nm spectral range using a Triax 180 Jobin Yvon monochromator with an R5108 Hamamatsu PMT (HAMAMATSU PHOTONICS DEUTSCHLAND GmbH, Herrsching am Ammersee, Germany). The PL signal was recorded in both cases through an SRS lock-in amplifier (SP830 DPS; Stanford Research Systems, Inc., Sunnyvale, CA, USA) referenced to the chopping frequency of light of 9.6 Hz. All PL spectra were corrected on the spectral response of experimental setup.

## Results and discussion

### Photoluminescence spectra

**Figure 1:**
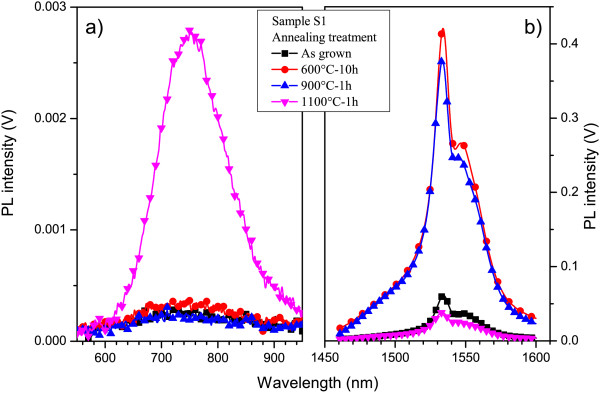
**Photoluminescence spectra.** Photoluminescence spectra of the sample detected for as-grown and annealed samples in (**a**) visible spectral range (500 to 950 nm) and (**b**) infrared spectral range (1.4 to 1.6 *μ*m). The experiments have been carried out using the 476.5-nm wavelength (nonresonant excitation for Er^3+^ ions).

The PL spectra, recorded on the as-deposited layer and after different annealing treatments, are reported in Figure 1. The highest PL intensity in the 500- to 950-nm spectral range is detected for the sample annealed at 1,100°C for 1 h (Figure 1a). This PL band is a feature of Si-ncs, which confirmed the Si-nc formation in our sample similar to the results of another work [21]. In the infrared spectral range (1.4 to 1.6 *μ*m), the highest Er^3+^ PL efficiency was obtained for the sample annealed at 600°C (Figure 1b). Meanwhile, the increase of annealing temperature from 600°C to 900°C results in the slight decrease of the Er^3+^ PL emission. Further temperature rise from 900°C to 1,100°C leads to a decrease of the PL intensity by a factor of 10 (Figure 1b). By comparison, the PL efficiency at 1.53 *μ*m of the as-deposited layer is slightly higher than that observed for 1,100°C annealed sample. Based on previous results [12,13], this behavior of Er^3+^ emission in as-deposited layer suggests that Si sensitizers are already formed, allowed by the relatively high deposition temperature (500°C). Another argument for Si-nc formation is the absence of Er^3+^ emission in Er-doped SiO_2_ counterparts submitted to the same annealing treatment. To explain the lowering of the Er^3+^ PL intensity after 1,100°C annealing, APT experiments have been performed on the as-deposited and 1,100°C annealed samples.

### Atom probe experiments

**Figure 2:**
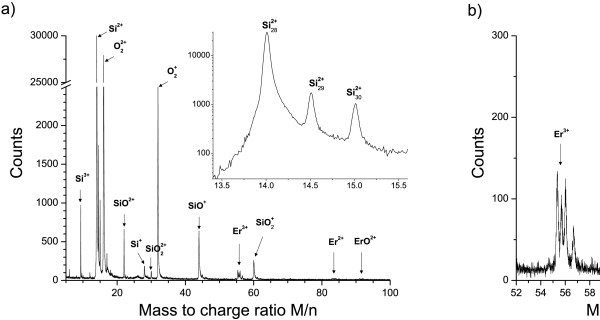
**Atom probe mass spectrum.** APT mass spectrum obtained on Er-doped Si-rich SiO_2_ sample. (**a**) Typical mass spectrum with Si, O, and Er identified peaks. Isotopes of silicon for the Si^2+^ peak are evidenced in the inset. (**b**) Magnification of the Er peaks in the 52- to 96-M/n region.

Prior to the study of microstructure, chemical analysis of the samples was performed by means of the APT technique. A typical mass spectrum of Er-SRSO layers is shown in Figure 2. The mass-over-charge ratio is a characteristic of the chemical nature of each ion collected during atom probe analysis. The presence of the three chemical elements (Si, O, and Er), constituting our samples, is clearly seen (Figure 2). Silicon is identified, after field evaporation, in three different charged states: Si^3+^, Si^2+^, and Si^1+^. The three isotopes of silicon are detected to be in good agreement with their respective relative natural abundances (Figure 2a). The oxygen is found as molecular ions $O_{2}^{2+}$ and $O_{2}^{1+}$ (Figure 2a). Finally, erbium ions are mostly detected as Er^3+^ or Er^2+^ (Figure 2b). The composition deduced from the mass spectrum of the as-grown and annealed samples is presented in Table 1. No significant difference of the overall composition can be seen for both samples analyzed. The Er content, measured as approximately 1.0×10^21^at/cm^3^, is in agreement with that expected from fabrication conditions [29].

**Table 1:** APT compositions of the Er-doped SRSO layer in the as-deposited and 1,100°C 1-h annealed state

	**As-deposited**	**Annealed at 1,100°C**
Si (at.%)	35.1 ± 0.4	35.0 ± 0.4
O (at.%)	63.2 ± 0.4	63.1 ± 0.4
Er (at.%)	1.7 ± 0.4	1.9 ± 0.4
Er (at·cm^−3^)	1.1 × 10^21^	1.3 × 10^21^
Si excess (at.%)	Approximately 3.6 %	Approximately 3.5%

**Figure 3:**
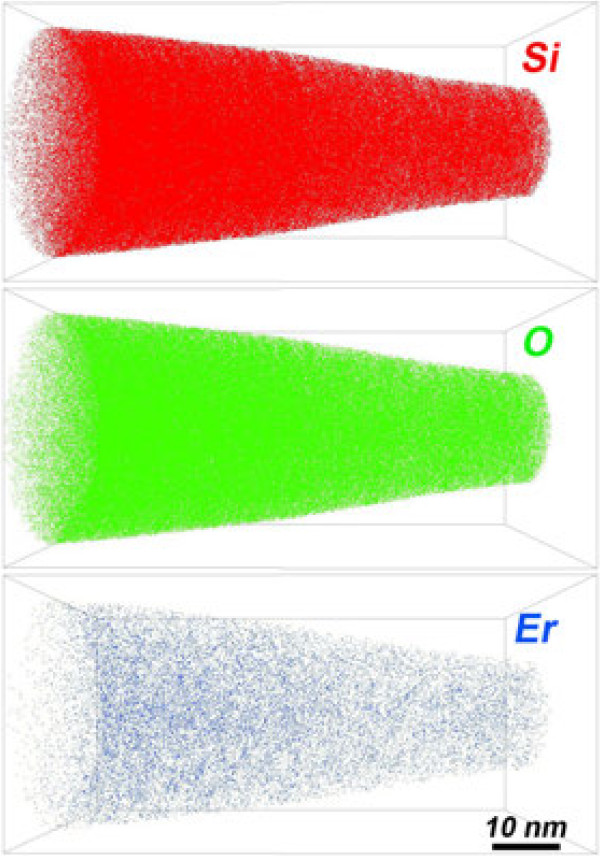
**3D reconstruction of the as-grown Er-doped SRSO layers of APT analysis.** APT reconstruction of 3D distribution of silicon, oxygen, and erbium atoms in the as-grown sample. The volume analyzed is 41×41×88 nm^3^ .

Figure 3 shows the 3D distributions of Si, O, and Er atoms within the reconstructed volume obtained from the APT analysis of the as-deposited layer where each dot corresponds to one atom detected. Statistical treatment of APT data was used to quantify concentration fluctuations in the sample. Frequency distribution was compared to binomial distribution to evidence the phase separation and atom clustering. This treatment performed on as-deposited material indicates a homogeneous spatial distribution of the three chemical species (Si, O, and Er) in the analyzed volume (41 × 41 × 88 nm^3^). Thus, it suggests that no Er clustering occurs during the deposition process. Moreover, it is worth to note that, based on these frequency distributions, we estimate that Si-ncs or Er clusters with a diameter below 0.8 nm (corresponding to agglomerated 15 Si atoms or 10 Er atoms) could not be distinguished from free Si or Er atoms. These atomic scale investigations, correlated with the PL data (Figure 1), suggest that in the as-deposited sample, the Si sensitizers consist of less than 15 Si atoms and are efficient to excite neighboring Er^3+^ ions.

**Figure 4:**
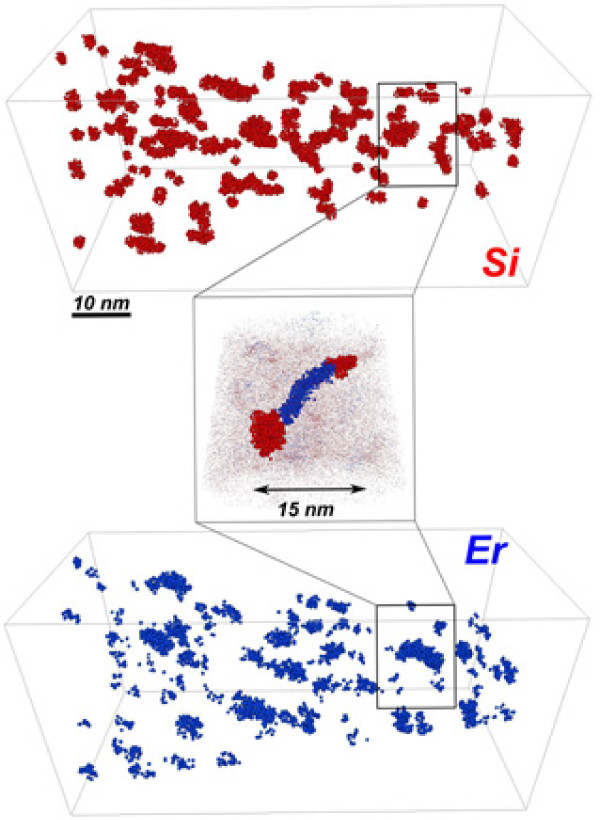
**3D reconstruction of the annealed Er-doped SRSO layers of APT analysis.** 3D cluster-filtered distributions of chemical species (Si in red and Er in blue) in Er-doped SRSO layers annealed at 1,100°C for 1 h. For clarity, only silicon and erbium atoms which belong to clusters are represented. Two Si-ncs linked by Er-rich aggregates are shown in the inset.

Before 2003 [13], the standard annealing treatment, applied for the formation of Si-NCs in Si-rich SiO_2_ materials fabricated by different approaches, was an annealing at 1,100°C for 1 h in pure nitrogen gas. The same annealing treatment was considered to be efficient to create the Si-NCs in Er-doped Si-rich SiO_2_ materials to achieve a sensitizing effect towards rare-earth ions. Figure 4 shows the 3D cluster-filtered distribution of chemical species in the Er-SRSO layers submitted to such thermal treatment. The Si-ncs are clearly seen; their density is estimated to be about (3.1 ± 0.2)×10^18^Si-ncs/cm^3^. The mean distance between Si-ncs, derived from their density, is found to be 6.9±0.2 nm, which is in agreement with that deduced from the 3D reconstruction. The Si-ncs are spherical in shape and are homogeneously distributed in the analyzed volume. Simultaneously, a large density of Er-rich clusters approximately (2.0×10^18^Er-NCs/cm^3^) has also been detected in the sample (Figure 4). Furthermore, some Si-ncs are interconnected by Er clusters (or channel) as illustrated in the inset of Figure 4. No particular morphology of these Er clusters has been deduced.

This result shows that thermal treatment at 1,100°C leads to a formation of a three-phase system: silica matrix, Si-ncs, and Er-rich clusters. The formation of such Er clusters is accompanied by the enlargement of the distance between Si-ncs, and it explains why annealing at 1,100°C quenches the PL emission with respect to the lower annealing treatments. Although the formation of Si-ncs increases the probability of absorbing excitation light, the total number of Si sensitizers decreases due to the merging of several small Si sensitizers along with the increase of Si-to-Er distance.

The measurement of the clusters’ composition, which can be difficult in APT volume, has been performed using the procedure developed by Vurpillot et al. [30] and was recently applied by Talbot et al. on similar Si nanostructured materials [18,25]. The size distribution of the Si-ncs is well fitted by a Gaussian law. The minimum and maximum observed radii are 0.9 ± 0.3 and 2.3 ± 0.3 nm, respectively, whereas the mean radius of Si-ncs was estimated to be <*r*>=1. ± 0.3 nm. Along with this, about 50% of Si-ncs have the radii in the range of 1.0 to 1.5 nm. The volume fraction of Si clusters is given by the following formula:

(1) $$f_{V}^{\text{Si}}=\frac{C_{\text{Si}}^{0}-C_{\text{Si}}^{M}}{C_{\text{Si}}^{C}-C_{\text{Si}}^{M}},$$

where $\left(C_{\text{Si}}^{C}\right)$, $\left(C_{\text{Si}}^{0}\right)$, and $\left(C_{\text{Si}}^{M}\right)$ are the compositions of Si in the Si-pure clusters, in the whole sample and in the matrix, respectively. The compositions have been extracted from the concentration (in at.%) using the density of pure Si (*d*_Si_=5.0×10^22^ at./cm^3^) and pure silica (*d*__SiO2__=6.6×10^22^ at./cm^3^); $\left(f_{V}^{\text{Si}}=4.0\right)$% is obtained from Equation 1.

**Figure 5:**
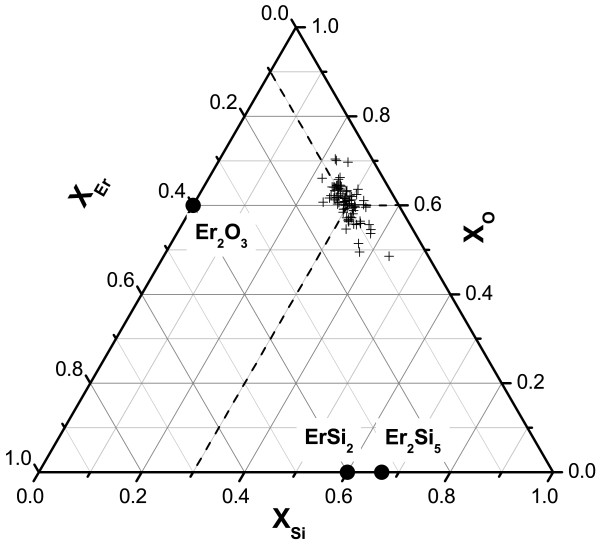
**Composition of erbium rich clusters.** APT composition measurements of individual Er-rich clusters compositions reported in the ternary Si-O-Er phase diagram.

The Si diffusion coefficient has been deduced from the Einstein equation of self-diffusivity: $<\rho >=\sqrt{6\text{Dt}}$, where < *ρ* > is the average displacement in three dimensions, *t* is the diffusion time, and *D* is the diffusion coefficient. The average displacement < *ρ* > was estimated as the mean distance between the surfaces of two first- neighbor Si-ncs. The Si diffusion coefficient at 1,100°C, deduced from our data (< *ρ* >=4.3 nm and *t*=3,600 s) is equal to *D*_Si_=8.4×10^−18^ cm^2^/s. Such a value is close to the silicon diffusion coefficient measured in Si-implanted SiO_2_ materials (*D*_Si_=5.7×10^−18^ cm^2^/s) obtained by Tsoukalas et al. [31,32]. As far as the Er-rich clusters are concerned, we have reported all the measured compositions of individual cluster on the ternary phase diagram Si-O-Er (Figure 5). This figure clearly illustrates that the composition of Er-rich clusters deals with a non-equilibrium phase in comparison with ErSi_2_, Er_2_Si_5_, or Er_2_O_3_ expected from the binary equilibrium phase diagram of Er-Si and Er-O. Moreover, the present results are consistent with those of Xu et al. [33] and Kashtiban et al. [34], who have showed the absence of the mentioned Er equilibrium compounds in similar Er-doped Si-rich SiO_2_ materials. The mean composition of Er-rich clusters is $\left(X_{\text{Er}}^{C}=11.9\pm 0.4\right)$ at.%, $\left(X_{O}^{C}=59.7\pm 0.4\right)$ at.% and $\left(X_{\text{Si}}^{C}=28.4\;\pm \;0.4\right)$ at.% which corresponds to the ErSi_3_O_6_ phase. This result is also in agreement with similar ErGe_*X*_O_*Y*_amorphous clusters observed recently by Kanjilal et al. by HRTEM [35]. The volume fraction ($\left(f_{V}^{\text{Er}}\right)$) and atomic fraction ($\left(f_{a}^{\text{Er}}\right)$) of Er atoms in the clusters are given by the following formula (assuming the same density between Er-rich clusters and silica matrix):

(2) $$f_{V}^{\text{Er}}=\frac{C_{\text{Er}}^{0}-C_{\text{Er}}^{M}}{C_{\text{Er}}^{C}-C_{\text{Er}}^{M}},$$

(3) $$f_{a}^{\text{Er}}=\frac{C_{\text{Er}}^{C}}{C_{\text{Er}}^{0}}\left(\frac{C_{\text{Er}}^{0}-C_{\text{Er}}^{M}}{C_{\text{Er}}^{C}-C_{\text{Er}}^{M}}\right),$$

where $\left(C_{\text{Er}}^{C}\right)$, $\left(CX_{\text{Er}}^{0}\right)$ and $\left(C_{\text{Er}}^{M}\right)$ are the compositions of Er in the Er-rich clusters, in the whole sample and in the matrix, respectively. Following Equations 2 and 3 , the atomic and volume fractions are estimated to be $\left(f_{a}^{\text{Er}}=30\right)$% and $\left(f_{V}^{\text{Er}}=4.8\right)$%. This indicates that after annealing, about 70% of the total Er amount remains in solid solution as ‘isolated’ atoms, whereas the rest (30%) of Er^3+^ ions belongs to Er-rich clusters. We should note that the content of Er atoms, detected in our sample after 1,100°C annealing step, exceeds the solubility limit of Er in SiO_2_, estimated as 0.1 at.% (<10^20^ at/cm^3^) [36,37]. This explains the decrease in the Er^3+^ PL emission noticed in this film (Figure 1) after such a high-temperature annealing treatment similar to that reported in another work [29]. Moreover, we can note that the decrease of the PL intensity is higher than expected if only 30% of the Er amount is located in Er-rich clusters. To explain such a decrease, we assume that annealing treatment leads to the Si-nc density decreases (while Si-nc size increases) and the increase of Si-nc-Er interaction distance as well as to the decrease of the number of optically active Er ions coupled with Si-ncs.

The 3D chemical maps also indicate that the Er-rich clusters are likely formed in the vicinity of Si-ncs upon an annealing stage. This fact can be attributed to a preferential segregation of Er atoms at the Si-ncs/matrix interface during the phase separation process, similar to the results reported by Crowe et al. [38]. However, this hypothesis is not supported by the results of Pellegrino et al. [11], who concluded to a preferential segregation of Er in poor Si-nc region. In their paper, a double-implantation annealing process was applied to fabricate an Er-doped SRSO layer. This double process may stimulate Er diffusion explaining the segregation of Er and Si during the different implantation stages, which is contrary to our case.

Based on the hypothesis of spherical radius and on the determination of an amount of Er, Si, and O atoms in Er-rich clusters detected by APT method, the mean Er-rich cluster radius is estimated to be 1.4 ± 0.3 nm in the sample annealed at 1,100°C (<  *ρ*  >=5.1 nm and *t*=3,600 s). Erbium diffusion coefficient in the SRSO layer has been deduced using the Einstein equation of self-diffusivity. It has been found to be *D*_Er_≈1.2×10^−17^cm^2^· s ^−1^ at 1,100°C. This value is about one order of magnitude lower than that reported by Lu et al. (4.3×10^−16^cm^2^· s ^−1^) [39] which has been measured in SiO_2_. This difference could be attributed to the presence of Si excess in the film.

The formation of Er-rich clusters explains the evolution of the optical properties of Er-doped layers upon high-temperature annealing treatment applied [12,13,29]. It is worth to note that the fabrication approach, chemical composition, and microstructure of initial samples define strongly the effect of post-annealing processing.

## Conclusions

In this paper, the first investigation by APT, to our knowledge, of the nanostructure of Er-doped silicon-rich silica layer was performed at the atomic level and correlated with photoluminescence properties. The phase separation process between Si excess and the surrounding matrix was studied, and a formation of Si-rich or Er-rich phases was observed for samples annealed at high-temperature (1,100°C). The Si excess atoms precipitate in the form of pure Si nanoclusters in the silica matrix. Simultaneously, Er atoms form Er-rich clusters (about 30% of total amount), whereas 70% of the total Er atoms are free-dispersed in the host, demonstrating a super-saturation state but with an increase of the Si-ncs-to-Er distances. The Er-rich clusters have complex shape and composition. They are localized at the Si-nc/matrix interface or in poor Si-nc regions, indicating a complicated precipitation mechanism. Diffusion coefficients for Si and Er have been deduced from APT experiments. We have directly evidenced the clustering of rare-earth ions upon high-temperature annealing in Er-doped Si-rich SiO_2_ films. This process has been often expected but, to our knowledge, never observed and demonstrated directly for these materials fabricated by different techniques. These results evidence the critical point to monitor the microstructure of Er-doped SRSO layers for the required inversion of 50% of the Er concentration to achieve a net gain in future Er-doped amplifier device.

## Competing interests

The authors declare that they have no competing interests.

## Authors’ contributions

ET and RL carried out the APT sample preparation by SEM-FIB and performed the atom probe analysis and data treatment. ET, LK, and FG wrote the paper. FG, LK, and KH fabricated the sample under investigation and carried out the optical measurements. PP supervised the study and made significant contributions to the structural properties. All authors read and approved the final manuscript.
